# The Impact of Overweight and Obesity on Plantar Pressure in Children and Adolescents: A Systematic Review

**DOI:** 10.3390/ijerph17186600

**Published:** 2020-09-10

**Authors:** Liliana Catan, Elena Amaricai, Roxana Ramona Onofrei, Calin Marius Popoiu, Emil Radu Iacob, Corina Maria Stanciulescu, Simona Cerbu, Delia Ioana Horhat, Oana Suciu

**Affiliations:** 1 Department of Rehabilitation, Physical Medicine and Rheumatology, “Victor Babes” University of Medicine and Pharmacy Timisoara, Eftimie Murgu no 2, 300041 Timisoara, Romania; catan.liliana@umft.ro (L.C.); onofrei.roxana@umft.ro (R.R.O.); oanasuciu78@umft.ro (O.S.); 2 Department of Paediatric Surgery, “Victor Babes” University of Medicine and Pharmacy Timisoara, Eftimie Murgu no 2, 300041 Timisoara, Romania; mcpopoiu@umft.ro (C.M.P.); radueiacob@umft.ro (E.R.I.); stanciulescu.maria@umft.ro (C.M.S.); 3 Department of Radiology, “Victor Babes” University of Medicine and Pharmacy Timișoara, Eftimie Murgu no 2, 300041 Timisoara, Romania; cerbusimona@yahoo.com; 4 Department of ENT, “Victor Babes” University of Medicine and Pharmacy Timișoara, Eftimie Murgu no 2, 300041 Timisoara, Romania; deliahorhat@yahoo.com

**Keywords:** plantar pressure, overweight, obesity, children, adolescents

## Abstract

We aimed to synthesise the results of previous studies addressing the impact of overweight and obesity on plantar pressure in children and adolescents. An electronic search of scientific literature was conducted using PubMed, Cochrane and Scopus database, with keywords: “plantar pressure” AND “children” AND “obesity”; “plantar pressure” AND “adolescents” AND “obesity”, “plantar pressure” AND “children” AND “overweight”, “plantar pressure” AND “adolescents” AND “overweight”. Twenty-two articles were included in the review and the following data were recorded: authors, publication year, type of technology (systems, software) for the determination of plantar pressure, study characteristics. Most of the articles used dynamic plantar pressure determination with only four using static plantar pressure measurement. Using ultrasonography with static plantar pressure determination, the correlation between structural and functional changes in the feet of obese children. In overweight and obese children and adolescents, important findings were recorded: higher contact area, increased maximum force beneath the lateral and medial forefoot, increased pressure–time integral beneath the midfoot and 2nd–5th metatarsal regions. Significantly increased foot axis angle and significantly flatter feet were observed in obese subjects in comparison to their normal-weight counterparts. The obese children presented increased midfoot fat pad thickness, with decreased sensitivity of the whole foot and midfoot.

## 1. Introduction

In children and adolescent obesity has become a growing challenge and a concern of the worldwide public health services. It is a disease with an increasing prevalence and long-term medical and socioeconomic consequences. At adulthood, obese children and adolescents have a high risk of cardiovascular diseases, diabetes and other comorbidities.

The World Health Organisation (WHO) reported all over the world more than 340 million children and adolescents aged 5–19 years in 2016 and 50 million children younger than five years in 2018 that were overweight or obese. According to the WHO, overweight and obesity are defined as abnormal or excessive fat deposits that can affect health status. For the school-aged children and adolescents (aged between 5 and 19 years), overweight is defined as the body mass index for age greater than one standard deviation over the WHO growth reference standard median; for obesity, body mass index for age is more than two standard deviations over the WHO growth reference standard median. For children younger than five years, overweight is the weight for height greater than two standard deviations above the median of WHO standards for child’s growth; obesity is defined as more than three standard deviations above the median of WHO standards for child’s growth [1]. In 2010, it was estimated that the prevalence of overweight and obesity in children will be 14.1% in 2020, in comparison with that of 7.9% in 1999 [2].

In children and adolescents, overweight determines a burden on the musculoskeletal system. Through its compensatory demand, it can produce effects on the posture and the entire biomechanical body axis. It can also affect the mobility, physical activity and performance of age-specific everyday activities, as well as musculoskeletal pain issues in different parts of the body. Obesity has an impact on foot structures, with changes in the anatomical structures, an abnormal distribution of the plantar pressure and balance problems [3,4,5,6].

The foot overload and the changes in plantar pressure parameters due to obesity have an impact on children and adolescents, drawing attention to medical professionals and researchers in the last two decades. Scientific studies have sustained these findings by using various tools for the evaluation of plantar pressure [6,7,8,9,10]. The last one is a method of assessing the interaction between foot posture and lower limb biomechanics; by analysing its parameters, the evaluation of the interaction with the entire lower limb can be achieved [11]. There is a variability of foot structure and plantar pressure depending on age and type of determination (static or dynamic) [12], but also depending on the number of assessments according to age group [13] or the type and level of physical activity. In children and adolescents, a strong inverse correlation was reported between the plantar pressure and the level of physical activity [14].

The evaluation of plantar pressure can signal different foot postural changes. It can also provide data related to forces at the foot level while standing and walking in both healthy subjects and in different pathologies such as obesity. The plantar pressure offers information regarding changes due to pain complaints at the level of the lower limb [15], stability disorders or falls [16]. In children and adolescents, the gain in body weight determines plantar pressure increases; this limits the foot functionality and can cause pain and discomfort with the middle foot area being the most susceptible [15].

The aim of our review was to analyse the results of previous studies addressing the impact of overweight and obesity on the plantar pressure parameters in children and adolescents. The analysis consists of a narrative qualitative synthesis systematising the conclusions of the included studies and making suggestions related to future research. This will include both the assessment and the decision-making in order to prevent the long-term musculoskeletal complications due to a body mass excess, to promote the necessary physical activity, establish a rehabilitation programme and follow its efficiency.

## 2. Materials and Methods

We conducted an electronic search of the scientific literature using the PubMed, Cochrane and Scopus database, as well as the references of the selected studies. The keywords were: “plantar pressure” AND “children” AND “obesity” (35 articles); “plantar pressure” AND “adolescents” AND “obesity” (9 articles), “plantar pressure” AND “children” AND “overweight” (29 articles), “plantar pressure” AND “adolescents” AND “overweight” (8 articles). The search was not limited to publication year; we included the studies published prior to March 2020. The English language was accepted as a criterion. The abstracts were not taken into account.

The approval of the ethics committee was not necessary. The systematic review does not contain data that violate human rights, as included in the Declaration of Helsinki.

**Figure 1:**
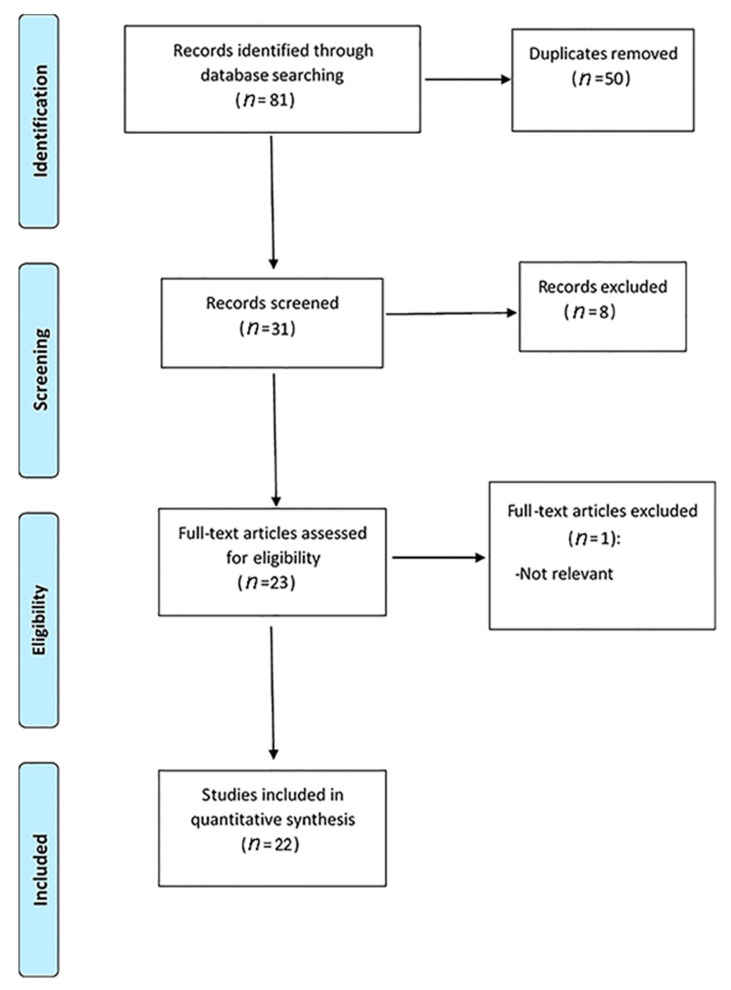
Flowchart of study selection.

A first record of the research was achieved from the PubMed, Cochrane and Scopus database using the abovementioned keywords; 81 studies were included. Fifty studies were excluded due to their repetition; the titles and abstracts of the remaining 31 studies were exported in an Excel file. Two investigators read and assessed the recordings. Furthermore, they excluded the studies that did not involve children or adolescents with obesity (3 studies), the studies that had not obesity as an inclusion criterion (1 study) and those that did not have plantar pressure as an assessment tool (4 studies; Figure 1).

In the next phase of the study, the remaining 22 articles were the subject of the narrative synthesis. Each article was analysed and the following data were recorded: authors, publication year and month and country where the research was conducted, type of technology (systems, software) for the determination of plantar pressure, study characteristics (PICOS: P—Patient, Problem or Population, I—Intervention, C—Comparison, Control or Comparator, O—Outcome(s), S—Study type [17,18], size of the study, follow-up period), particularities of each study with the estimated effects, main findings and limitations. Table 1 presents the summary of the studies included in the analysis of the current paper: number of participants, group comparison, type of plantar pressure system and method for determining plantar pressure, main variables of the plantar pressure and significant findings.

Twenty-two publications from 9 countries (Australia: 36.36%, Italy: 18.18%, Brazil: 13.63%, China: 9.09%, USA: 4.54%, Spain: 4.54%, Germany: 4.54%, UK: 4.54% and Israel: 4.54%) met the inclusion criteria; they were of interest for the complete text examination in the process of the final review.

## 3. Results

### 3.1. Plantar Pressure during Walking/Running

A recent controlled nonrandomised study, published in March 2020 by Molina-Garcia et al. [19], within the project “MUévete Bien” (MUBI), describes the effects of a 13-week tailored physical exercise programme on plantar pressure in overweight or obese children while walking. The conclusions of the research pointed out the importance of adding a tailored exercise programme in children with obesity. The exercises should be based on the quality of analytic movements, with the acquisition of fundamental movement abilities. The last ones can have a role in the structural and functional changes in plantar pressure, as well as in the foot dynamics during walking with a positive impact on the walking model in adolescent and future adults.

These observations are similar to those of the study of Mesquita et al. [20] that concluded that in obese children the plantar pressure distribution during running was modified (Table 1). The results of this study showed that while running the obese children generated more forces when compared with those with lower weight (whole foot maximum force of 597.98 ± 26.49 N in normal weight compared with 704.77 ± 44.17 N in overweight and 873.66 ± 54.86 N in obese, *p* < 0.050). The body mass index (BMI) was positively correlated with peak pressure for the whole foot (*r* = 0.340; *p* = 0.027), midfoot (*r =* 0.550; *p* < 0.001) and forefoot (*r =* 0.454; *p =* 0.003). They had a larger contact area under all foot areas (*p* < 0.050). Thereby, this study highlights that obesity is associated with the increase of plantar pressure during running with self-selected speeds (speeds identified as the average walking/running speed of five overground trials on a 10 m route [21]. Moreover, it issued the hypothesis that obese children have a higher risk of developing foot discomfort and pain.

The plantar pressure distribution during different movements (natural comfortable walking used in everyday activities, slow running/jogging and fast running—50 m race) is distinctive in obese children in comparison to those with a normal weight, as noticed by the study of Song-Hua et al. [22].

The authors noted that in obese children the propulsion phase during jogging was the longest of the three subphases; in nonobese children, the longest propulsion phase was during fast running. When compared to the nonobese group the obese group showed a shorter propulsion phase during walking (42.37% ± 8.26% versus 48.17% ± 10.80%) and running (47.82% ± 6.53% versus 52.56% ± 8.32%). During jogging, obese children had a longer propulsion phase (48.27% ± 5.16%) in comparison to nonobese children (48.19% ± 7.91%). In obese children, the peak pressures corresponding to the fourth and fifth metatarsal heads, midfoot, medial and lateral heel during jogging were the highest for all the three movements. In these subjects, the highest arch index was for the left foot, while jogging. The study concluded that in obese children, the peak pressures in most of the plantar regions and the arch index have the most significant values while jogging. Thus, jogging causes increased stress at lower limbs. This category of subjects (with obesity) should not consider jogging as a common exercise.

In 2013, the same authors published the results of a study that investigated the effects of obesity on plantar pressure distribution while walking; The results showed that obese subjects had longer midstance duration (left foot: 49.45 ± 7.72%; right foot: 49.36 ± 7.90%, compared to nonobese left foot: 43.06 ± 10.60%; right foot: 45.37 ±10.03%) and shorter propulsion duration (left foot: 41.81 ± 7.80%; right foot: 41.68 ± 8.27%), compared to nonobese subjects (left foot: 46.20 ± 9.17%; right foot: 44.69 ± 8.43%). The peak pressures under the 2nd–5th metatarsal heads, midfoot and heel lateral were significantly higher for obese subjects. The time to peak pressures under the 4th and 5th metatarsal heads, and midfoot, and pressure rate under the heel, medial and lateral heel were also significantly increased. The authors observed that the stability while walking was weaker and the dynamic distribution of plantar pressure changed in obese children when compared to nonobese children. The obese children had a flat foot model, with an enlarged angle of the foot axis [23].

In children, overweight leads to a higher total load with a disproportionate impact on the midfoot and its longitudinal arch; these are the representative areas of foot loading as revealed by the study of Mueller et al. [24]. The authors presented detailed information for the significant differences in the dynamic characteristics of the foot in normal weight, overweight and obese children, for each age group ranging from 1 to 12 years. The following data were recorded: contact area, arch index, peak pressure and force–time integral for the whole foot, for the anterior, mid and posterior parts. The average speed was 0.95 ± 0.25 m/s, without differences among normal weight, overweight and obese children. The results of the study showed that the foot contact area, arch index, maximum pressure and force–time integral of the force–time had higher values in overweight and obese children (*p* < 0.001). In obese children, the midfoot loading was 1.48 times higher in the one-year group and 3.49 times higher in the 10-year group in comparison to normal-weight subjects. The research found that the feet of children aged 1–2 years were significantly affected; overweight and obesity were not compensated by the musculoskeletal system.

Another descriptive study, published in 2014, describes the impact of physical activity on foot and plantar pressure of overweight and obese children. After six months, they noted the followings: a significant decrease in BMI *z*-score for the physical activity group (2.36 ± 0.61 compared with 2.62 ± 0.60, *p =* 0.001) and in the group without physical activity (2.22 ± 0.67 compared with 2.65 ± 0.68, *p* < 0.002), an increase of the internal arch height in the physical activity group (24.0 ± 1.3 mm compared with 23.2 ± 1.3 mm) and no physical activity group (23.1 ± 0.8 mm compared with 21.8 ± 1.1 mm), with no change in total physical activity. Internal arch height is the distance from the supporting surface of the platform to the joint beneath the dorsal navicular landmark (measured at the same site as the fat pad thickness measurement). Pressure–time integrals increased (lateral and medial midfoot, and lateral and medial forefoot in the physical activity group). However, after a six-month physical activity programme, there were no differences in plantar pressure parameters between the two groups. The study concluded that the changes in foot structure and function in overweight and obese children could not be assigned to the physical activity programme [25].

The same research team published in 2015 the results of a controlled randomised study on 73 overweight and obese children (age 8.3 ± 1.1 years; 47 girls and 26 boys; BMI *z*-score = 2.7 ± 0.7). The dynamic distribution of the plantar pressure was determined with the same system, as mentioned above, a calibrated Emed AT-4 pressure system (25 Hz, four sensors per square centimetre; Novel Gmbh, Munich, Germany). Each subject wore an ActiGraph 7164 accelerometer (ActiGraph, Pensacola, FL, USA) during an 8 h period of walking hours, except for the aquatic activities. The correlation coefficients of the Pearson moment result were calculated in order to determine the power of the relations between the maximum plantar pressure during walking and the physical activity levels. The peak pressures generated beneath the forefoot during walking were inversely correlated with time spent performing activities of different intensity levels. Moderate-intensity (*r =* −0.321, *p* = 0.007), vigorous-intensity (*r =* −0.326, *p* = 0.006), and moderate- to vigorous-intensity (*r =* −0.342, *p* = 0.004) physical activity were significantly correlated with middle forefoot pressure and with lateral forefoot pressure (*r =* −0.248, *p* = 0.040; *r =* −0.264, *p* = 0.028; *r =* −0.267, *p* = 0.027, respectively). Lateral midfoot (*r =* −0.244, *p* = 0.044) and second toe (*r =* 0.227, *p* = 0.021) pressures were also significantly correlated with vigorous-intensity activity. The conclusions were that children with higher pressures under the forefoot and midfoot while walking had lower levels of physical activity. The authors suggested that further studies are required in order to determine the long-term effects of overweight. The patterns of plantar pressure associated with low physical activity should be studied in relation to pain and discomfort at the foot level [26].

In 2013, the results of a study on 100 children showed that overweight seven-year-old children had differences in foot loading while walking when compared to normal-weight subjects. The results showed that obese and overweight children had significantly higher peak pressures, peak forces, force–time and pressure–time integrals under the midfoot and 2nd–5th metatarsal regions. After the normalisation of peak force, the obese and overweight subjects demonstrated significantly greater loading at the midfoot and 2nd–5th metatarsals. The authors proposed an early assessment and intervention in overweight and obese children for the prevention of musculoskeletal complications due to excessive body mass [27].

In order to answer the question if the excess of body mass affects plantar pressure in young children while walking, Mickle et al. (2006) [28] conducted a study on preschool children. The results showed that, in comparison to normal-weight children, when walking, the overweight and obese children had significantly higher contact areas and generated important forces on the plantar surface of the whole foot, heel, midfoot and forefoot. In spite of these increased forces on higher contact areas, the overweight and obese participants had higher peak pressures, time–force integrals and pressure–time integrals at midfoot in comparison to normal-weight children. Although the overweight and obese children had an increased contact on the foot surface, this contact area was not sufficient to compensate the raised forces while walking, leading to increased plantar pressures in comparison to children without excess body mass. The force–time integrals displayed by overweight/obese children below the middle leg region (10.19/5.6 N·s) were significantly higher than those displayed by nonobese children (5.49/4.1 N·s). The overweight/obese children recorded higher pressure–time integrals in the midfoot region (29/0.7 N·s/cm^2^) than their nonoverweight counterparts (1.59/0.5 N·s/cm^2^). These results suggested that their midfoot can be exposed to increased stress and can be vulnerable to bone overload and to soft tissue deterioration. The authors recommended that further investigations should be carried out, as their observations are addressed not only to children, but also to adults. The changes they noticed were disorders in foot structure and function, pain and physical activity, with implications for the children’s growing period.

Steinberg et al. in 2017 [21] investigated the influence of a weight-reduction programme with a locomotive emphasis on improving biomechanical characteristics of 30 overweight children. There were five types of examinations—walking at 80%, 100% and 120% of the typical walking velocity and ran at 80% and 100% of the typical running velocity on the treadmill. The second group had improved foot loading during walking and running when compared with the other groups. The second group had no significant change in BMIPs but improved their biomechanical characteristics and the first group with a decrease in BMIPs had no improvement in gait characteristics. Body mass index percentiles (BMIP) was used to classify the children in a weight category. Overweight was considered as a BMIP more than 85, while normal weight was a BMIP 85 or less, according to normative international age and gender BMI cut-offs for children. They reported a significant decrease in total plantar surface area, maximum force and force integrals in children with both obesity management and locomotion-emphasis programme. The authors concluded that the gait improvement in children who are overweight is related to the specific gait exercise and not to weight loss.

The programme proposed by Steinberg et al. [21] was two times longer than the one of Molina-Garcia et al. [19], suggesting that longer exercise programmes could be necessary for force reductions at the foot level. The increase of maximal forces on the forefoot and foot dynamics during walking and running determines changes to a more adult gait pattern, namely a medially loaded foot [20]. Although the changes in foot structure and function in overweight and obese children cannot be attributed to the attendance of physical exercise programmes, increased plantar pressure and pressure–time integrals can lead to foot pain [25].

In obese children, sustained physical activity, based on the quality of analytic movements and the acquisition and practice of fundamental movement abilities can lead to positive structural and functional changes in foot dynamics during walking. Physical activities lead to an increase in maximum force beneath forefoot and to a plantar surface area increase. In the abovementioned category of subjects (overweight and obese), self-selected speed running and jogging led to a raise of maximal pressures in most of the foot areas and a change in the foot arch index. These variations can determine the overloading of the lower limbs, clinically expressed by pain and discomfort [20,22,25]. Children with overweight and obesity have a longer propulsion phase when jogging that increases the risk of foot injury; they should not practice jogging as a regular exercise. Because of higher peak pressures under lateral forefoot, midfoot and rearfoot while jogging in comparison to fast running, jogging may cause foot problems [22]. Overweight children with reduced daily physical activity generate higher plantar pressures at forefoot and midfoot when compared to their normal-weight counterparts and those overweight with increased physical activity [26].

The dynamic distribution of plantar pressure raises, with a maximum at forefoot (2nd–5th metatarsi) and midfoot (especially the medial part) [23,24,27,28,29]. Moreover, the foot arch index [23,24] and foot axis angle [23] have larger values. The evaluation of static plantar pressure shows that overweight and obese children generate higher forces on a higher foot area and increased plantar pressures when compared to their normal-weight counterparts, especially for the midfoot and under the heads of 2nd–5th metatarsi [30,31].

### 3.2. Plantar Pressure during Balance Test

A randomised study included 11 obese children (six boys and five girls, mean age 11.4 ± 1.2 years, height 1.59 ± 0.09 m, weight 64.3 ± 13.8 kg) and 11 nonobese children (six boys and five girls; mean age 11.5 ± 2.3 years, height 1.51 ± 0.16 m, weight 43.2 ± 13.9 kg) who performed two 30 s static balance tests (eyes open followed by eyes closed) on a plantar pressure distribution system (EMED-SF; Novel GmbH, Munich, Germany). The tests showed that both groups had a higher persistence for small oscillations; the effect was superior for obese children. That was obvious with eyes closed, with significant differences for the two groups for reduced oscillations. The study concluded that balance disturbances in obese children are caused by sensorial disorders and by a lower proprioceptive ability [8].

### 3.3. Static and Dynamic Plantar Assessments

A study from 2008 [32] aimed to evaluate if, in obese children, the static footprints can predict the plantar pressure. That was due to the fact that static footprints offer only indirect information about the height of the medial longitudinal foot arch, especially in obese children. Thus, the authors drew the conclusions that in obese children the relation between the static and dynamic measurements is affected. They suggested that decision-making should not consist just of the use of footprints and podobarography.

Another study published in 2004 hypothesised that obese children have a high risk of foot discomfort and foot disorders due to the increased plantar loadings on a growing skeletal structure. These ideas have been sustained by Mickle et al. in their previously mentioned study [28]. The results of the static assessment showed that obese children generated significantly higher forces on both left and right leg (698.17 ± 247.7 N and 688.77 ± 228.8 N compared with 440.27 ± 142.1 N and 348.47 ± 125.4 N) on a higher foot area (86.37 ± 21.2 cm^2^ and 87.07 ± 21.6 cm^2^ compared with 60.17 ± 13.0 cm^2^ and 54.27 ± 12.1 cm^2^). When compared to normal-weight children they also had increased values of plantar pressure (41.8 ± 17.7 vs. 30.1 ± 12.0 N/cm^2^, *p* < 0.022), midfoot and second head metatarsal head plantar pressures. The authors noticed that obese children seemed to flatten the midfoot during walking.

In an 11-year period (between 2000 and 2011), Pau, et al. [30] examined 118 children and adolescents with Down syndrome. The results showed that the excess of body mass influences significantly the foot–ground contact and generates an increased plantar pressure in mid and forefoot regardless of their sex (midfoot +26%; forefoot +32%, for the males; midfoot +33%; forefoot +37% for the females). There were gender-related differences, with girls having higher contact areas and increased plantar pressures in normal-weight subjects. Flat foot was prevalent in both groups, whereas its incidence does not seem to have a connection with obesity.

One important parameter is the force–time integral; it provides information regarding the load on particular foot structures. An increased force–time integral in overweight/obese children suggests that the midfoot area or the longitudinal arch structure may be exposed to stress. Pressure–time integral has also higher values in overweight/obese children and is frequently associated with soft tissue damage [19,25,28]. In the current review, the most-used foot anthropometric parameters were the arch index (high values recorded in all the studies in obese compared with normal-weight children) and the plantar arch height (low values recorded in all articles in obese compared with normal-weight children). The plantar arch height was measured from the supporting surface to the lowest medial foot protrusion at the instep landmark.

### 3.4. Temporary Mass Increases Versus Long-Term Mass Increase and Plantar Pressure

The effects of obesity on plantar pressure distribution have been studied in 26 prepubescent children. They were divided into two homogeneous groups: 13 obese children (age 8.1 ± 1.2 years, BMI 25.5 ± 2.9 kg/m^2^) and 13 normal-weight children (age 8.4 ± 0.9 years, BMI 16.9 ± 1.2 kg/m^2^). The footprints were recorded using a podograph with calculation of Chippaux–Smirak index. The children underwent static and dynamic plantar pressure analysis using a mini-Emed 33 system (Novel GmbH, Munich, Germany). An additional 20% loading was added by wearing a waistcoat both in static and dynamic determinations. The authors noted that a rapid increase of body mass influences the static and dynamic plantar pressure parameters in comparison to the long-term effects of obesity. They noted a smaller footprint angle (*t* = 4.107; *p* < 0.001) and higher values of Chippaux–Smirak index (*t* = −6.176; *p* ˂ 0.001) in obese children in comparison to normal-weight children. These structural changes in the foot have been associated with differences in plantar pressure between the two groups. The peak dynamic pressure values in obese subjects (39.3 ± 15.7 N/cm^2^) were significantly higher than those generated in the normal-weight children (32.3 ± 9.2 N/cm^2^). The conclusions of this study were that structural changes associated with foot discomfort and increase in plantar pressure can limit the obese children to engage in physical activities [29].

Two studies included in the current review assessed the effect of schoolbag wear on plantar pressure distribution and forces exchange between ground and body in overweight and obese children. The first study of Pau, et al. [33] included 65 overweight and obese primary school students (32 boys and 33 girls) and 65 normal-weight students, age, gender and height-matched. The plantar pressure parameters while walking was determined in both groups with and without wearing the schoolbag. The study observations are relevant for the prevention of long-term possible side effects regarding foot structure and functionality. This can also be useful in establishing adequate limits for schoolbags in school students [33].

Another study of Pau et al. [34], having the same abovementioned objectives, included two groups of children, homogeneous in age. This study, opposed to the previous one that included also a dynamic assessment, determined the static plantar pressure. The children were firstly assessed with a schoolbag as in a usual school day; afterwards, they were assessed without wearing the schoolbag. Overweight and obese children had larger contact areas and higher peak plantar pressures compared with the normal-weight group (significantly higher peak plantar pressures in the rearfoot and midfoot by 17% and 37% in boys and 38% and 27% in girls with no significant increase in the forefoot). In overweight and normal-weight participants, the backpack induced a similar generalised increase in contact area and pressures. Obese boys had significantly higher peak pressures in the midfoot and forefoot by 13% and 22%, respectively, versus normal-weight boys and obese girls who had increased peak pressure values for all of the plantar subregions with the largest effect observed in the forefoot −20% increase. The results showed that the greatest changes were at the forefoot, suggesting that the loading tends to modify the models of physiologic plantar pressure.

An important aspect that causes discomfort in overweight and obese children is carrying additional weight. When added on the trunk (for example a schoolbag), it changes the plantar pressure parameters, mostly at the forefoot, both while standing [34] and walking [33]. This is important for establishing ergonomic measures and for setting up limitations of the trunk load carried by children and adolescents [33,34].

### 3.5. Plantar Pressure and Fat Pad Thickness

In two studies, Riddiford-Harland et al. [35,36] investigated the relation between the fat thickness of the obese children’s foot and the distribution of static and dynamic plantar pressure. The results sustained the effects of obesity on the children’s feet during growth. In their first study [35], the authors aimed to determine if the flat foot structure in school-age children is due to the fat thickness of the midfoot or is secondary to the structural changes through the drop of the foot longitudinal arch. The thickness of the plantar fat pad directly was measured beneath the joint between the inferior aspect of the navicular and middle cuneiform. The authors noticed that obese children had significantly higher medial midfoot fat pad thickness relative to the leaner children during both non-weight-bearing (5.4 and 4.6 mm, respectively; *p* < 0.001) and weight-bearing (4.7 and 4.3 mm, respectively; *p* < 0.001). The obese children also displayed a lowered medial longitudinal arch height when compared to controls (23.5 and 24.5 mm, respectively; *p* = 0.006). The study stated that the flat foot and the increased midfoot fat pad thickness in obese children does not have clinical and functional relevance.

The authors extended their research in the following study trying to establish a relation between obesity and the distribution of plantar pressure parameters in movement. Results showed that both medial midfoot plantar fat pad thickness and medial midfoot plantar pressure were correlated with BMI (*r* = 0.401, *p* < 0.001 and *r =* 0.465, *p* < 0.001, respectively). The medial midfoot plantar pressure significantly correlated with midfoot plantar fat pad thickness during non-weight-bearing (*r =* 0.294, *p* < 0.001) and weight-bearing (*r =* 0.289, *p* < 0.001); however, the strength of the correlations was weak. The authors concluded that in overweight children, the midfoot fat pad is only the consequence of the excessive body mass rather than the adjustment of plantar pressure [36].

Similarly to the two previous studies, Mickle et al. [37] in 2006 published the results of a study that was focused on the determination of the mechanism of flat foot in small children with overweight and obesity. The authors questioned if the flat foot in these children was due to the thicker midfoot fat pad or to the drop of foot longitudinal arch reported to normal-weight subjects. They found no significant difference comparing the thickness of the midfoot plantar fat pad recorded for the overweight/obese children (4.3 ± 0.6 mm) with the values recorded for the nonoverweight children (4.1 ± 0.6 mm). The results showed no significant between-group differences in the thickness of the midfoot plantar fat pad. However, the overweight and obese children had a significantly lower plantar arch height (0.9 ± 0.3 cm) than their nonoverweight counterparts (1.1 ± 0.2 cm). The authors concluded that the drop of plantar arch height and the flat foot in overweight children can be caused by the foot structural changes, with a negative impact on long-term functionality if the body mass excess persists over time.

### 3.6. Plantar Pressure and Foot Sensitivity

The results of Da Rocha et al. [38], published in 2014, were similar to those previously mentioned. They stated that in obese children, the plantar pressure is increased. They also brought into discussion a less studied aspect, namely the foot sensitivity in this category of children. Their results showed that, due to overweight, subjects had diminished foot sensitivity and a similar sensitivity in certain foot areas in comparison to gender, age and height-matched normal-weight subjects. The results were compared between obese and nonobese participants, between different foot regions and between the right and left foot. The conclusions suggested that due to an increased plantar pressure and reduced foot sensitivity, obese children are predisposed to a higher risk of foot injury, with limitation in activity performance.

### 3.7. Plantar Pressure and Type of the Foot

A study published by Cimolin et al. [39] in 2016 strengthened the idea that, in adolescents, obesity has an impact on plantar pressure distribution, but also on the foot type and structure. The analysis showed that obese participants had a significantly higher contact area on the forefoot and midfoot (medial region) when compared to controls. No differences were recorded for the posterior part of the foot. Regarding the maximum pressure and force, the results were similar for both groups. Obese participants had increased values for all the regions, except for the back middle part that showed similar results in both groups. As regards the foot classification in obesity adolescents, the authors noticed that 70% had flat foot, 20% cavus foot and 10% a normal foot type. In contrast, 25% of the normal-weight group had flat foot, 25% cavus foot and 50% a normal foot type.

Considering the plantar pressure assessment tools, a number of 14 studies used a dynamic plantar pressure system, while seven studies used a static plantar pressure system. Only one article [39] utilised an in-shoe system; the rest of the studies used external pressure platforms. Most articles focused on dynamic plantar pressure, analysing contact areas, peak and mean pressures under different anatomic areas of the foot, pressure–time and force–time integral. Peak pressure in overweight/obese children progresses from the midfoot area in preschool period to midfoot and forefoot in primary-school-age and to midfoot, forefoot and heel in obese adults. Due to different types of pressure platform (sensors/cm^2^ and acquisition frequency) there were slight differences among the studies [19,20,22,23,24,25,26,27,31,32,36,39,40]. There were also differences regarding the contact areas. Some pressure systems software divides the foot areas into 10 anatomical regions, others only in three anatomical regions. However, the results are similar regardless of these differences.

## 4. Discussion

The current review synthesises the findings related to an important topic for medical practice, namely excess body mass in children and adolescents, as well as its impact on plantar pressure.

In children, the excess of body mass determines a disproportion among the static and dynamic plantar pressure parameters in comparison to the normal-weight subjects; the last ones have a correlation among the abovementioned parameters [32]. This fact should be taken into account in the clinical practice. Without time correction, changes in plantar pressure distribution can impact physical activity performance and prevent children from taking part in age-specific physical activities due to pain and discomfort [28,29]. In overweight and obese children, an early assessment of plantar pressure distribution is necessary for the prevention of foot structural changes and functional complications [27,28]. This evaluation is also important when structuring a physical exercise programme for these subjects.

The most important deformation of the foot in obese children is the flat foot [41]. The flat foot and a thicker fat pad of the midfoot are consequences of structural changes due to the drop of foot longitudinal arch, as noticed in static [36,37] and dynamic assessments [36]. These alterations reflect the excess of body mass rather than the adjustments of plantar pressures [35,36].

It was noticed that, in obese subjects, the ground contact foot area increases during regular walking. A greater plantar surface area in overweight and obese children can be partially explained by the prevalence of flat foot [41]. A larger fat mass may reflect a greater presence of deformable soft tissues in the plantar region, which, under the action of increased load, determines larger contact areas [30]. Additional body mass leads to a higher whole foot load, especially for the midfoot and longitudinal foot arch.

The stability of overweight and obese children while walking is affected due to a flatter foot pattern [23,31]. The loading is higher at the midfoot starting from the moment of acquisition of the standing posture and walking; it increases with the child’s growing period. The mechanic overstress due to an excess of body mass cannot be compensated by the musculoskeletal system [24]. Balance control is essential for daily life, representing the basis for most movements that children perform. In overweight and obese children balance dysfunctions can be present as a result of a reduced proprioceptive ability and sensorial disorders [8]. This can affect the long-term therapeutic decisions such as the inclusion of overweight and obese children in physical exercise programmes. The objectives of physical exercise are not only weight reduction and correction of musculoskeletal deviations, but also the proprioception and balance improvement. Thus, these children will have a lower risk of injuries and better movement and game abilities due to enhanced coordination and stability. After the literature analyses, we point out the need for further research in order to investigate physical activity methods for the reduction of plantar pressure parameters in overweight and obese children.

The obese children can have a lower sensitivity in different parts of the foot in comparison to normal-weight subjects, predisposing thus to a higher risk of lower foot injuries [38]. Excessive stress on the plantar region leads to reduced plantar sensitivity consequently to continuous hyperactivation of mechanoreceptors [42].

Most articles focused on comparing the plantar pressure in normal-weight and overweight/obese children. The majority of articles were made on school-aged children; only one study included adolescents. We found no study to compare the parameters of plantar pressure distribution, maximal forces, peak pressures and foot contact areas among overweight/obese children and overweight/obese adolescents. A subject of interest can be the group age analysis, starting from the early child standing period and carried out during the preschool and school age periods, preadolescence and adolescence. When comparing the overweight subjects starting from the age of 1 until 12, it was recorded that the loading increases with age for all the foot areas, in particular for the forefoot and midfoot [24]. However, there are no data registered for the 12–18-year groups.

Although the analysed studies also included the subjects’ gender, a comparison of gender differences was not performed. Separate analyses for boys and girls would be of interest because gender differences in foot morphology might also reflect the foot–ground interaction. A study on obese children with Down syndrome showed that contact pressure was found significantly higher in the midfoot and the forefoot of those with obesity, regardless of their gender [30]. In another research regarding the influence of supplementary weight-bearing in obese children, Pau et al. found no significant differences among girls and boys without wearing a backpack. With additional weight-bearing (backpack) some differences were observed: overweight/obese boys had significantly higher peak pressures in the midfoot and forefoot, and girls had increased peak pressure values for all of the plantar subregions, with the largest effect observed in the forefoot [34]. Future research should also include comparative analysis of gender and age groups in overweight and obese children and adolescents.

Another important aspect would be the evaluation of plantar pressure in overweight/obese children before and after weight loss. In 2007, Teasdale et al. [42] evaluated two groups of 14 obese (age 37.97 ± 7.7 years) and 14 morbid obese (age 44.47 ± 8.9 years) men before and after weight loss, for a 12-month period. They showed that weight loss had a beneficial impact on the overall postural stability of obese men, benefits correlated with the amount of weight loss. The authors concluded that this improvement may arise from smaller plantar contact areas with weight loss, allowing the mechanoreceptors to better detect postural oscillations.

After analysing the studies included in the current review, we point out that they are well structured; the methods used for plantar pressure evaluation varied and were adapted to technology. The assessment of plantar pressure in overweight and obese children and adolescents should represent a concern not only for medical doctors and researchers, but also for public health caretakers. The prophylaxis of this increasingly common disease (obesity), as well as an early diagnosis of musculoskeletal deformities, will have a long-term effect on the general health status, bone mineral density and on the delay of osteoarthritis disease, especially concerning hip, knee and spine [43]. The obese children and adolescents should be managed by an interdisciplinary team consisting of nutritionist, paediatrician, paediatric orthopaedics, rehabilitation specialist, physical therapist and psychologist.

We are aware that our review has limitations. Some of the studies included a relatively small number of patients. We took into research only articles, without including PhD theses, monographs or scientific books. Although the studies contained many variables recorded in overweight and obese subjects, the current review focused only on plantar pressure determinations in a limited group of subjects, namely children and adolescents.

## 5. Conclusions

This paper reviewed a group of articles reported in the PubMed database with similar aspects regarding the plantar pressure determination in obese and overweight children and adolescents. The most frequently used plantar pressure parameters were peak pressure, maximum force, contact area, pressure–time integral and structural foot parameters (arch index and angle of the foot axis). In overweight and obese children and adolescents, important findings were recorded: higher contact area, increased maximum force beneath the lateral and medial forefoot, increased pressure–time integral beneath the midfoot and 2nd–5th metatarsal regions. Significantly increased foot axis angle and significantly flatter feet were observed in obese subjects in comparison to their normal-weight counterparts. The obese children also presented increased midfoot fat pad thickness, with a decreased sensitivity of the whole foot and midfoot. We conclude that besides the clinical examination, the assessment of plantar pressure in overweight and obese children and adolescents should be also considered in order to determine the early changes in foot structure and function.

## Figures and Tables

**Table 1:** Summary of included studies.

Authors/Country/Year and Month of Publication	Number of Participants	Group Comparison	Plantar Pressure System	Method for Determining Plantar Pressure	Plantar Pressure Variables	Significant Findings (Group Comparisons)
Fink et al./USA/2019 April [8]	22 children with O/NW	*Group one:* 11 children with O (height 1.59 ± 0.09 m, weight 64.3 ± 13.8 kg) *Group two:* 11 children with NW (height 1.51 ± 0.16 m, weight 43.2 ± 13.9 kg)	Plantar pressure distribution system (EMED-SF; Novel GmbH, Munich, Germany)	*Static balance tests* (Two 30 s static balance tests: eyes open followed by eyes closed)	Displacement of centre of pressure (COP); Euclidean distance	*Group one: * ↑ Persistence for small oscillations ↓ Proprioceptive ability
Molina-Garcia et al./Spain/2020 March [19]	70 children with O/OW	*Group one:* 39 children (performed 13-week exercise programme BMI 25.59 (25.2 to 25.99)) *Group two:* 31 children (had a usual lifestyle) BMI 25.44 (25.1 to 25.79)	Pressure platform FreeMed^®^ Pro (Sensormedica, Rome, Italy) with 450,000 pressure sensors	*Dynamic plantar pressure measurement* (Barefoot—Plantar pressure distribution and loading while walking)	Contact area (CA); maximal force (MF); force–time integral (FTI) 11 anatomical regions (lateral and medial rearfoot, lateral and medial midfoot; 1st, 2nd, 3rd, 4th and 5th metatarsal; hallux; 2nd to 5th toes)	*Group one: * ↑ MF, specifically under the lateral and medial forefoot *Group two:* ↑ CA ↑ FTI forefoot
Mesquita et al./Brazil/2018 May [20]	42 children with O/OW/NW	*Group one:* 9 children with O (BMI = 22.90 ± 0.7 kg/m^2^) *Group two:* 14 children with OW (BMI = 19.21 ± 0.42 kg/m^2^) *Group three:* 19 children with NW (BMI = 15.23 ± 0.22 kg/m^2^)	Pressure platform Emed AT-4 (Novel GmbH, Munchen, GE; 50 Hz; 4 sensors/cm^2^; 415 × 255 mm)	*Dynamic plantar pressure measurement* (Barefoot—During running 5–10 min with a self-selected speed);	Maximum force; normalised maximum force; contact area; peak pressure under each anatomic region (6 anatomical regions: whole foot, rearfoot, midfoot, forefoot, hallux and lesser toes)	*Group one: * - Generated more forces, except for the hallux - BMI was positively correlated with PP for whole, midfoot and forefoot - ↑ CA under all foot areas
Nili Steinberg et al./Israel/2017 October [21]	30 children with O	*Group one:* 10 children (Dietary Intervention during the 6-month) (BMI 25.74 ± 3.07 kg/m^2^) *Group two:* 10 children (a similar programme as group one, with additional specific exercises, in a twice-weekly training session for 1 h each) (BMI 24.25 ± 1.79 kg/m^2^) *Group three:* 10 children (with no intervention programme) (BMI 25.13 ± 4.76 kg/m^2^)	A portable insole system (Novell Pedar, Munich, Germany)	*Dynamic plantar pressure measurement* (3 different velocities of walking and 2 different velocities of running x group × pre-/postintervention) 6 areas: heel, inner medial, lateral medial, inner forefoot, lateral forefoot, and toes	Contact area; length of contact; length of contact percentile; peak pressure; Maximum force; foot pressure–time integral and Foot force–time integral	*Group one:* ↓ CA in walking, ↑ PP, ↑ MaxF *Group two:* *↓* CA, ↓ PP, ↓ MaxF, *Group three:* ↑ CA, ↓ PP in walking and ↑ PP in running, ↓ MaxF in walking
Song-Hua et al./China/2017 September [22]	40 children with O/NW	*Group one:* 20 children with O (BMI 28.13 ± 3.40 kg/m^2^) *Group two:* 20 children with NW (BMI 17.44 ± 1.57 kg/m^2^)	Plantar pressure mat (RSscan International, Belgium)	*Dynamic plantar pressure measurement* (Barefoot—During three movements: natural comfortable walking used in everyday activities, slow running, such as jogging and fast running; 50 m race)	Plantar pressure (at 10 anatomic regions); subphases of stance phase; peak pressure; arch index and angle of the foot axis	*Group one* has the most significant values while jogging: ↑ PP in most of the plantar regions ↑ AI
Song-Hua et al./China/2013 January [23]	100 prepubescent children with O/NW	*Group one:* 50 children with O (BMI of 23.68 ± 3.00 kg/m^2^) *Group two:* 50 children with NW (BMI of 17.08 ± 1.25 kg/m^2^)	The system of foot scan plantar pressure (RSscan International, Olen, Belgium), with a plate of 0.578 m/0.418 m, 4096 resistive sensors and a resolution of 4 sensors/cm^2^	*Dynamic plantar pressure measurement* (Barefoot—Plantar pressure distribution and loading while walking)	Subphases during foot–ground contact duration; peak pressures; time to peak pressures and pressure rate in 10 plantar regions; foot arch index; relative regional impulses (RIR) under three plantar regions; foot balance and foot axis angle	*Group one:* ↑ Midstance duration ↓ Propulsion duration ↑ AI for the left foot, and the left and right foot axis angle ↑ PP under the 4th and 5th metatarsal heads, and midfoot ↓ Stability while walking - Have a flat foot model
Mueller et al./Germany/2016 February [24]	7575 Children with O/OW/NW	*Group one:* 371 children with O (BMI 23.1 ± 3.3 kg/m^2^) *Group two:* 746 children with OW (BMI 19.7 ± 1.9 kg/m^2^) *Group three:* 6458 children with NW (BMI 16.4 ± 1.5 kg/m^2^)	Pressure platform (Emed X1, Novel GmbH, Munich, Germany), mounted in the walkway	*Dynamic plantar pressure measurement* (Barefoot—Plantar pressure distribution and loading while walking)	Contact area; arch index (AI); force–time integral; peak pressure 5 anatomical regions: toes, forefoot, medial midfoot, lateral midfoot and hindfoot	*Group one and two:* ↑ CA ↑ AI ↑ Peak pressure midfoot and forefoot ↑ Force–time integral of the force–time *Group one compared to group three:* the midfoot loading was 1.48 higher in the 1-year group and 3.49 higher in the 10-year group
Riddiford-Harland et al./Australia/2016 January [25]	34 children with O	*Group one:* 24 children (performed a 10-week physical activity programme) *Group two:* 10 children (10 children did not practice any physical activity) (BMI *z*-score for all: 2.63 ± 0.61)	AT-4 Emed system (Novel GmbH, Munich, Germany) and ActiGraph	*Dynamic plantar pressure measurement* (Barefoot—Plantar pressure distribution while walking)	Mean peak pressure footprints; peak pressure; pressure–time integral 10 anatomical regions	*Group one* and *two:* ↓ BMI (*z*-score) ↑ Foot length; ↑ Foot height; ↑ PTI—lateral midfoot and forefoot - No differences in plantar pressure parameters between the two groups
Riddiford-Harland et al./Australia/2015 February [26]	73 children with O/OW	*One group:* 73 children with O/OW (BMI *z*-score = 2.7 ± 0.7)	AT-4 Emed (25 Hz, 4 sensors/cm^2^; Novel GmbH, Munich, Germany) and ActiGraph 7164 accelerometer (ActiGraph, Pensacola, FL)	*Dynamic plantar pressure measurement* (Barefoot—Plantar pressure distribution while walking)	Mean peak pressure footprints; peak pressure; 10 anatomical regions	PP generated beneath the forefoot during walking were inversely correlated with time spent in different intensity levels of physical activity
Cousins et al./UK/2013 August [27]	100 children with O/OW/NW	*Group one:* 22 children with O (BMI = 24.16 ± 3.14 kg/m^2^) *Group two:* 22 children with OW (BMI = 19.17 ± 1.28 kg/m^2^) *Group three:* 56 children with NW (BMI = 15.63 ± 2.04 kg/m^2^)	MatScan^®^ 3150 5 m platform (TekScan, USA) Portable ultrasound (SonoSite ^®^ 180 PLUS system, Washington, USA) with a linear transducer (10–5 MHz, maximum depth of 7 cm)	*Dynamic plantar pressure measurement* (Barefoot—Plantar pressure distribution while walking)	Peak pressure; peak force; normalised peak force; pressure–time integral; force–time integral. (At six regions of the plantar foot—lateral heel, medial heel, midfoot, 1st metatarsophalangeal joint, 2nd–5th metatarsophalangeal joint and hallux)	*Group one* and *two:* Under the midfoot and 2nd–5th metatarsal regions: ↑ PP, ↑ PTI, ↑ FTI
Mickle et al./Australia/2006 [28]	34 children with O/NW	*Group one:* 17 children with O (BMI 18.59 ± 1.3 kg/m^2^) *Group two:* 17 children with NW (BMI 15.79 ± 0.7 kg/m^2^)	The pressure platform AT-4 (25 Hz; Novel GmbH, Munich, Germany)	*Dynamic plantar pressure measurement* (Barefoot—Plantar pressure distribution while walking)	Peak pressure; maximum force; maximum contact area; Pressure–time integral and force–time integral. (heel, midfoot, forefoot, hallux and toes 2–5)	*Group one: * ↑ PP, ↑ CA of the whole foot, heel, midfoot and forefoot ↑ PTI and ↑ FTI in the midfoot region,
Dowling et al./Australia/2001 January [29]	26 children with O/NW	*Group one:* 13 children with O (BMI 25.5 ± 2.9 kg/m^2^) *Group two:* 13 children with NW (BMI 16.9 ± 1.2 kg/m^2^)	One podograph and mini-Emed1 system (Novel GmbH, Munich, Germany)	*Static and dynamic plantar pressure measurement* Barefoot	Fingerprint angle and Chippaux–Smirak index; peak static and dynamic force; peak static and dynamic area; peak static and dynamic pressure. (For the whole foot). Dynamic rearfoot and forefoot force. 2 anatomic regions	*Group one:* ↓ footprint angle ↑ Chippaux–Smirak index ↑ Peak dynamic forefoot pressures ↑ Peak forces ↑ Peak contact area ↑ Forefoot contact area
Pau et al./Italy/2013 October [30]	118 children and adolescents (with Down syndrome) with O/OW/NW	*Group one:* 59 children and adolescents with O/OW (BMI 26.7 ± 3.9: male, 28.3 ± 3.9: female kg/m^2^) *Group two:* 59 children and adolescents with NW (BMI 19.6 ± 4.0: male, 18.0 ± 2.1: female kg/m^2^)	A pressure-sensitive carpet (Tekscan Inc, South Boston, MA) consisting of 2016 detection elements embedded in a 42 × 48 matrix	*Static plantar pressure measurement* Barefoot	Contact area (total, rearfoot, midfoot, forefoot); arch index; peak plantar pressures (rearfoot midfoot, forefoot) 3 anatomic regions	*Group one:* ↑ CA (girls only)) ↑ PP (midfoot +26%, and forefoot +32%, for the males; midfoot +33%, and forefoot +37%, for the females) *Group one* and *two:* flat foot is the prevalent arch type
Dowling et al./Australia/2004 November [31]	20 children with O/NW	*Group one:* 10 children with O (BMI 25.8 ± 3.8 kg/m^2^) *Group two:* 10 children with NW (BMI 16.8 ± 2.0 kg/m^2^)	Pressure platform AT-4 Emed (Novel GmbH, Munich, Germany) with 4 sensors per cm^2^	*Static and dynamic plantar pressure measurement* Barefoot	Peak force; peak area; peak pressure; (for the total foot); force–time integral; pressure–time integral 10 anatomical regions	*Group one:* ↑ PF, ↑ PA, ↑ PP especially midfoot, ↑ contact area ↑ Forces over all areas of their feet, except the toes ↑ Values of plantar pressure, midfoot and 2nd head metatarsal ↑ PTI in lateral midfoot, forefoot - Flatten the midfoot
Taisa Filippin et al./Brazil/2008 November [32]	20 children with O/NW	*Group one:* 10 children with O (BMI = 28.4 ± 2.7 kg/m^2^) *Group two:* 10 children with NW (BMI = 15.8 ± 1.9 kg/m^2^)	Digital planimeter (Placom -CST) and Pedar system (Novel GmbH, Munich, Germany)	*Static and Dynamic plantar pressure measurement* (Barefoot—The static and dynamic distribution of the plantar pressure while walking on a 10 m walkway)	Arch index; static contact area (SCA); dynamic contact area (DCA); dynamic peak pressure (DPP); dynamic maximum mean pressure (DMMP).	*Group two:* correlations between the static and dynamic plantar pressure parameters ↓ Arch index: obese Dynamic midfoot area was greater than the static one for both
Pau et al./Italy/2016 May [33]	130 children with O/OW/NW	*Group one:* 65 children with O/OW (BMI 21.4 ± 2.3 kg/m^2^) *Group two:* 65 children with NW (BMI 16.7 ± 1.6 kg/m^2^)	A 4 m walkway with an embedded plantar pressure platform (FDM-S, Zebris Medical GmbH, Germany) was used; the platform had 2560 capacitive sensitive elements in a 64 x40 matrix, with an acquisition frequency of 100 Hz	*Dynamic plantar pressure measurement* (Barefoot—Plantar pressure distribution while walking with and without wearing the schoolbag)	Contact areas; arch index; Peak and mean plantar pressures (3 anatomic regions: forefoot, midfoot and rearfoot)	*Group two:* ↑ Mean midfoot pressure *Group one:* ↑ Larger contact area—all regions ↑ Dynamic arch index ↑ Mean, peak pressure midfoot and forefoot With schoolbag—↑ mean peak in forefoot and midfoot
Pau et al./Italy/2013 July–August [34]	140 children with O/OW/NW	*Group one:* 70 children with O/OW (BMI 21.6 ± 1.9: boys, 21.3 ± 2.7: girls kg/m^2^) *Group two:* 70 children with NW (BMI 16.3 ± 1.4: boys, 16.7 ± 1.7: girls kg/m^2^)	Footscan 0.5 system (RSscan International, Olen, Belgium)	*Static plantar pressure measurement* (Barefoot—Static plantar pressure distribution with and without wearing the schoolbag)	Contact area (total, rearfoot, midfoot, forefoot); arch index; peak plantar pressures (rearfoot midfoot, forefoot) 3 anatomic regions	*Group one:* ↑ CA ↑ PP (in the rearfoot and midfoot by 17% and 37% in boys and 38% and 27% in girls with no significant increase in forefoot) ↑ arch index
Riddiford-Harland et al./Australia/2011 January [35]	150 children with O/NW	*Group one:* 75 children with O (BMI 25.2 ± 3.6 kg/m^2^) *Group two:* 75 children with NW (BMI 15.9 ± 1.4 kg/m^2^)	Portable ultrasound (SonoSite ^®^ 180 PLUS system, Washington, USA) with a linear transducer (10–5 MHz, maximum depth of 7 cm)	*Ultrasonography of the midfoot* Barefoot	The midfoot fat pad thickness in both non-weight-bearing and weight-bearing positions; Height of internal arch	*Group one:* ↑ Midfoot fat pad thickness ↓ Medial longitudinal arch height
Riddiford-Harland et al./Australia/2011 August [36]	252 children with O/OW	*One group:* 252 children with O/OW (BMI 19.1 ± 4.3 kg/m^2^)	Portable ultrasound (SonoSite ^®^ 180 PLUS system, Washington, USA) with a linear transducer (10–5 MHz, maximum depth of 7 cm) and the pressure platform ^®^ Emed ^®^ AT-4 system (Novel GmbH, Munich, Germany).	*Ultrasonography of the midfoot and dynamic plantar pressure* Barefoot	The midfoot fat pad thickness in both non-weight-bearing and weight-bearing positions; Contact area; Force; Peak plantar pressure. (For the 10 anatomic foot regions)	- Medial midfoot plantar fat pad thickness and medial midfoot plantar pressure were correlated with BMI - Medial midfoot plantar pressure low correlated with midfoot plantar fat pad thickness during non-weight-bearing and weight-bearing
Mickle et al./Australia/2006 November [37]	38 preschool children with O/OW/NW	*Group one:* 19 children with O/OW (BMI 18.6 ± 1.2 kg/m^2^) *Group two:* 19 children with NW (BMI 15.7 ± 0.7 kg/m^2^)	Portable ultrasound system SonoSite 180 PLUS (SonoSite, Bothell, WA) with a large linear band of 38 mm, matrix transducer of 10–5 MHz, maximum depth of 7 cm). Pedograph (Suavepie, Capital Federal, Argentina)	*Ultrasonography of the midfoot* Barefoot	Arch index; plantar arch height; midfoot fat pad thickness in non-weight-bearing positions	*Group one:* ↓ Plantar arch height ↑ Arch index *Group one* and *two:* - No significant between-group differences in the thickness of the midfoot plantar fat pad
Da Rocha et al./Brazil/2014 August [38]	40 children with O/NW	*Group one:* 20 children with O (BMI 20.67 ± 1.78 kg/m^2^) *Group two:* 20 children with NW (BMI 16.27 ± 1.61 kg/m^2^)	Pressure aestheometry (Semmes–Weinstein Monofilaments, San Jose, USA) and the plantar pressure portable system (Matscan, Tekscan Inc., Boston, USA), with a sampling frequency of 100 Hz	*Foot sensitivity and static plantar pressure measurement* (During unipedal and bipedal stance, eyes opened and barefoot)	Sensitivity scores (Semmes–Weinstein pressure aesthesiometry); plantar pressure (whole foot, rearfoot, midfoot, forefoot) 3 anatomic regions	*Group one:* ↓ Foot sensitivity at whole foot and midfoot ↑ Plantar pressure for whole foot and all foot regions ↑ Pressure on rearfoot
Cimolin et al./Italy/2016 March [39]	18 adolescents with O/NW	*Group one:* 10 adolescents with O (BMI 35.45 ± 4.73 kg/m^2^) *Group two:* 8 adolescents with NW (BMI 18.67 ± 2.46 kg/m^2^)	The footwear system Pedar-X (Novel GmbH, Munich, Germany) in-shoe system	*Static plantar pressure measurement* In-shoe system—everyday sneakers corresponding to the individual’s size 8 anatomical regions (medial and lateral rearfoot, medial and lateral midfoot, hallux, medial central and lateral forefoot)	Peak pressure; peak force; contact area. Arch index (at medial and lateral rearfoot, midfoot; medial, central and lateral forefoot and hallux)	*Group one:* ↑ CA (on the forefoot and midfoot) - 70% had flat foot, 20% cavus foot and 10% normal foot type ↑ PP, ↑ PF for all the regions, with the exception of medial rearfoot area (similar between the two groups)

AI—arch index, BMI—body mass index, CA—contact area, COP—centre of pressure, DCA—dynamic contact area, DMMP—dynamic maximum mean pressure, DPP—dynamic peak pressure, FTI—force–time integral, LOC—length of contact, LOCper—length of contact percentile, MaxF—maximum force, MF—maximal force, NW—normal weight, O—obesity, OW—overweight, PA—peak area, PF—peak force, PP—peak pressure, PTI—pressure–time integral, RIR—relative regional impulses, SCA—static contact area.
